# Focal Cryotherapy in Prostate Cancer. Does Gleason Impact Results?

**DOI:** 10.1590/S1677-5538.IBJU.2025.0289

**Published:** 2025-12-20

**Authors:** Kinga Mate, Pedro de Pablos-Rodríguez, Marta Burbano Herraiz, Mario Hassi Román, Paula Pelechano Gómez, Ana Calatrava Fons, Maria Isabel Martín García, Jessica Patiño Aliaga, Manel Beamud Cortés, Álvaro Gómez-Ferrer Lozano, Jose Luis Dominguez Escrig, Cristina Gutierrez Castañé, Victor Rodríguez Part, Juan Luis Casanova Ramón Borja

**Affiliations:** 1 CHU de Pointe-à-Pitre Department of Urology Guadeloupe Pointe-à-Pitre France Department of Urology, CHU de Pointe-à-Pitre, Pointe-à-Pitre, Guadeloupe; 2 Péterfy Sándor utcai Hospital and Clinic Department of Urology Budapest Hungary Department of Urology, Péterfy Sándor utcai Hospital and Clinic, Budapest, Hungary; 3 Fundacion Instituto Valenciano de Oncologia Department of Urology Valencia Spain Department of Urology, Fundacion Instituto Valenciano de Oncologia, Valencia, Spain; 4 Hospital Universitario Miguel Servet Department of Urology Zaragoza Spain Department of Urology, Hospital Universitario Miguel Servet, Zaragoza, Spain; 5 Hospital DIPRECA Department of Urology department Santiago Chile Department of Urology department, Hospital DIPRECA, Santiago, Chile; 6 Fundación Instituto Valenciano de Oncologia Department of Radiology Valencia Spain Department of Radiology, Fundación Instituto Valenciano de Oncologia, Valencia, Spain; 7 Fundación Instituto Valenciano de Oncologia Department of Pathology Valencia Spain Department of Pathology, Fundación Instituto Valenciano de Oncologia, Valencia, Spain

**Keywords:** Prostatic Neoplasms, Cryotherapy, Prostate-specific antigen

## Abstract

**Purpose:**

Focal cryotherapy is a minimally invasive treatment for localized prostate cancer (PCa), but its oncological outcomes, particularly in relation to baseline Gleason Grade Group (GG), remain understudied. This study evaluates its efficacy and the impact while radical of baseline Gleason score on recurrence-free survival.

**Materials and Methods:**

A retrospective analysis included 111 patients with localized PCa treated with focal cryotherapy between 2014 and January 2024. Patients with prior treatments or follow-up <12 months were excluded. All patients underwent MRI and transperineal biopsy, and cryotherapy was performed using the Visual ICE Cryoablation System. Confirmatory biopsies were recommended at 12–24 months post-treatment. Recurrence was classified as either in-field (treated or adjacent areas) or out-field (non-adjacent areas). Any recurrence-free survival was defined as the absence of positive biopsy or additional treatment. Radical treatment-free survival was defined as the absence of whole-gland treatment (e.g., radical prostatectomy, radiotherapy), androgen deprivation therapy, metastasis, or death. Outcomes were compared between patients with baseline GG 1 and GG >1.

**Results:**

Median follow-up was 35 months (IQR 24–49). Confirmatory biopsies were performed in 78% of patients (n=87), revealing in-field recurrence in 10% and out-field recurrence in 23%. There were no statistically significant differences between ISUP 1 and ISUP >1 groups in terms of protocol biopsy positivity for either in-field recurrence (HR 0.41; 95% CI 0.09–1.9) or out-field recurrence (HR 0.77; 95% CI 0.3–1.98). At three-years, the rates of any recurrence-free and radical treatment-free survival were 63% and 85%, respectively, with no significant variation by baseline GG.

**Conclusion:**

Focal cryotherapy provides favorable short-term oncological outcomes in localized PCa, with no significant differences in recurrence-free survival based on baseline Gleason score.

## INTRODUCTION

Prostate cancer (PCa) is one of the most diagnosed malignancies in men worldwide. In Europe, it is the most frequently diagnosed cancer in men and ranks as the third leading cause of cancer-related mortality. The standard treatment options for patients with localized PCa are active surveillance (AS), radical prostatectomy (RP) or radiotherapy (RT). However, RP and RT are associated with significant morbidity, including urinary incontinence and erectile dysfunction, all of which can adversely impact quality of life (1). Additionally, active surveillance requires regular follow-up consisting of PSA testing, clinical examination, MRI imaging and repeated prostate biopsies (2, 3). More than one-third of patients are reclassified during follow-up, with the majority undergoing curative treatment due to disease progression (4). To enhance the benefit-to-risk ratio, alternative therapies have emerged that aim to minimize adverse effects while maintaining positive oncological outcomes (5, 6).

Focal cryotherapy, also known as cryoablation or cryosurgery, is a promising alternative for localized PCa. It enables targeted destruction of tumor tissue while preserving surrounding healthy structures. This technique induces apoptosis by the application of cryo-needles into the targeted area, leading to cell death via coagulative necrosis (7). The ideal candidate for focal cryotherapy remains uncertain. Patients with intermediate D’Amico risk with visible lesion in the MRI appear to be the primary candidates (8). Additionally, patients with low-risk disease but MRI-visible lesions have been reported to have worse oncological outcomes compared to those with non-visible lesions when initiating an active surveillance protocol (9). Furthermore, there is a lack of data comparing oncological outcomes based on patient's Grade Group (GG) Gleason score following focal therapy (FT). To our knowledge, there are no proven clinical factors, such as GG, to be used as indication for focal cryotherapy.

Several studies have highlighted the favorable functional outcomes of cryoablation, particularly, when compared to standard treatments (RP or RT) (10–12). However, oncological outcomes remain a critical area of investigation to determine the safety of this approach in managing localized PCa. Current guidelines from the NCCN (13) and EAU (14) recommend performing cryotherapy within prospective registries or clinical trials. To date, only a few centers have reported oncological outcomes following cryotherapy, and there is minimal evidence regarding GG and cryotherapy outcomes (15). Given the established prognostic value of Gleason score in PCa, we hypothesize that this variable impacts the likelihood of achieving disease control following focal cryotherapy.

In this study, we present our experience with short-term follow-up of patients treated with focal cryotherapy, focusing on the influence of baseline Gleason score.

## MATERIALS AND METHODS

This retrospective study included consecutive patients with primary localized PCa who underwent focal cryotherapy between 2014 and January 2024 at our institution. Exclusion criteria included previous prostate cancer treatments, suspicion of extra-prostatic disease, or follow-up shorter than 12 months. Patients were considered eligible if they had a single, histologically confirmed lesion in contiguous areas, whether visible or not on MRI. Factors such as age, PSA, prostate volume, high Gleason score, or severe LUTS were not considered exclusion criteria. Data were collected from a PCa registry (CAPROSIVO), which was approved by the local ethics committee.

All patients underwent preoperative MRI, with or without regions of interest (ROI), followed by transperineal biopsy. Most MRIs were performed at the Valencian Institute of Oncology using the General Electric Signa Artist 1.5 Tesla model. The images were interpreted by three experienced radiologists using the PI-RADS 2.0 or 2.1 version. For each ROI, 3-5 targeted biopsy cores were obtained, and systematic sextant biopsies (20 to 30 cores) were performed following a modified version of the Dickinson scheme, as previously described (16). Biopsies were conducted using the Hitachi V70 ultrasound system, with Biopsee software® (Medcom) used for fusion when required.

Cryotherapy was performed by the same experienced urologist (J.C.R) using the Visual ICE Cryoablation System (Boston Scientific). Patients were treated under general anesthesia with 2-4 IceSeed needles and were discharged the following day with a bladder catheter. The first visit took place 7–10 days after surgery, when the bladder catheter was removed. Follow-up visits were scheduled at 3, 6, and 12-months post-treatment, during which only PSA levels were measured. At 12 months, a multiparametric MRI was performed prior to the protocol biopsy. Beyond 12 months, patients underwent PSA testing every six months and MRI scans every 1 to 2 years to detect potential recurrence. Additional diagnostic procedures were reserved for cases with clinical suspicion of recurrence. Digital rectal examination was limited to the diagnostic phase and was not routinely employed during follow-up. No adjuvant androgen deprivation therapy (ADT) was used. Patients were advised to undergo a single confirmatory biopsy at 12–24 months after cryotherapy, unless recurrence was suspected earlier.

Regarding oncological outcomes, in-field recurrence was defined as any cancer foci within the previously treated area or directly adjacent regions. Adjacency was determined based on the transverse or craniocaudal sextants, excluding oblique or other sextants. Out-field recurrence referred to the detection of any cancer in non-adjacent areas of the prostate. Any recurrence-free survival was defined as the absence of a positive biopsy or any additional treatment at any time during follow-up. Radical treatment-free survival was considered as the absence of whole-gland treatment (brachytherapy, RT, RP), ADT, metastasis or death. Comparisons were performed between patients with baseline GG 1 vs GG >1, as well as according to baseline PSA level (≤6 vs >6 ng/mL) and PIRADS score (<3 vs ≥3).

### Statistical Analysis

Differences in categorical variables were assessed using chi-square tests, while differences in continuous variables were evaluated with t-test or Mann-Whitney U tests, as appropriate. The Log-Rank test and Kaplan-Meier curves were used to compare any recurrence and radical treatment-free survival across groups. All statistical analyses were performed using Python 3.13.0 software, with a significance level set at p <0.05.

## RESULTS

A total of 111 patients with localized PCa treated with focal cryotherapy were included. The median follow-up was 35 months (IQR 24-49). The median age at the time of cryotherapy was 70 years (IQR 64-74), and the median PSA was 6.3 ng/mL (IQR 4.6-8.6). As shown in Table-1, the majority of patients had non-palpable disease (91%) but visible lesions on MRI (80%).

**Table 1:** Baseline patients characteristics.

		Total (N=111)	GG 1 (N=40)	GG >1 (N=71)	P value
**Age, years**					
	Mean ± SD	68 ± 6.9	66 ± 7	70 ± 6.6	0.003
	Range	50–79	51–77	50–80	
**PSA, ng/mL**					
	Mean ± SD	7.2 ± 4.4	6.44 ± 3.18	7.5 ± 4.9	0.29
	Range	2.6–29	1.2–17	2.6–29	
**Clinical stage, n (%)**					0.39
	cT1c	101 (91)	39 (98)	63 (89)	
	cT2	10 (9)	1 (2)	8 (11)	
**Prostate volume, cc**					
	Mean ± SD	54 ± 26	57.4 ± 29	52 ± 24	0.39
	Range	18–142	18–142	19–126	
**MRI visible lesion, n (%)**		88 (80)	26 (65)	62 (87)	<0.05
**Grade Group, n (%)**					
	Grade Group 1	40 (36)	40 (100)	–	–
	Grade Group 2	55 (50)	–	55 (77)	
	Grade Group 3	13 (12)	–	13 (18)	
	Grade Group 4–5	3 (2)	–	3 (5)	
**Positive cores at initial biopsy**					
	Mean ± SD	3.3 ± 1.6	3.1 ± 1.7	3.3 ± 1.5	0.36
	Range	1–8	1–7	
**Positive millimeters at initial biopsy**					
	Mean ± SD	14 ± 11	13.6 ± 13.2	14.2 ± 9.3	0.27
	Range	0.6–58	2–49		

SD = Standard Deviation;PSA = prostate-specific antigen; MRI = magnetic resonance imaging; n = number of patients; GG = Grade Group; cc = cubic centimeters

At the end of the analysis, among the 111 patients in the cohort, 87 patients (78%) agreed to undergo a confirmatory biopsy, with a median time to biopsy of 18 months (IQR 14-19). The confirmatory biopsies revealed no cancer in 57 cases (66%), while 18 (21%) had Grade Group 1 disease, 8 (9%) had Grade Group 2 disease, and 4 (4%) had Grade Group >3 disease. Thus, 30 of these 87 patients (34%) had positive confirmatory biopsies, Grade Group ≥1 disease. In the entire cohort (111 patients), 36 patients experienced recurrence, defined as positive biopsy, radiological recurrence, or additional treatment, including four, identified by off-protocol biopsies and two by PSMA PET imaging. In-field recurrence was found in 10% of patients, while out-field recurrence was found in 23% of patients. There were no statistically significant differences between ISUP 1 and ISUP >1 groups in terms of protocol biopsy positivity for either in-field recurrence (HR 0.41; 95% CI 0.09–1.9) or out-field recurrence (HR 0.77; 95% CI 0.3–1.98). Patients who declined confirmatory biopsy had no clinical suspicion of recurrence, with a median PSA of 2 ng/mL (0.9-4.9) and negative MRI findings during follow-up.

**Figure 1:**
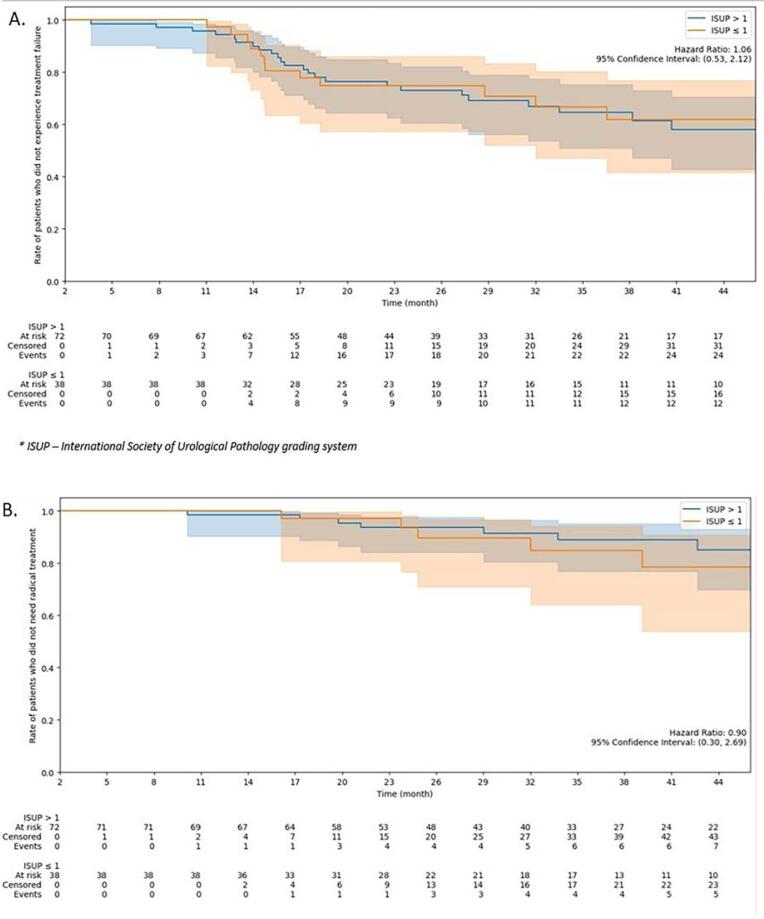
Kaplan-Meier curves by ISUP grade group (A) - Time to treatment failure (B) - Time to need for radical treatment.

Twenty (18%) of the 111 patients required secondary treatments, including brachytherapy (5 patients), second cryotherapy (7 patients), RT (2 patients), PT (2 patients), lymphadenectomy (1 patient) and ADT (3 patients). Radical treatments, excluding repeat cryotherapy and lymphadenectomy, were performed in 12 patients. At 3 years, 65% of patients were free from any recurrence, and 88% were free from radical treatment. As shown in Figure-1, no significant differences were observed between the initial GG 1 and GG > 1 groups regarding any recurrence-free survival (HR 1.2, 95% CI 0.6–2.5) or radical treatment-free survival (HR 1.1, 95% CI 0.35–3.2). Additionally, we compared recurrence-free survival according to baseline PSA levels (≤6 vs. >6 ng/mL) and PIRADS score (<3 vs. ≥3). No significant differences were observed in either analysis (HR 1.18, 95% CI 0.6–2.3 for PSA; HR 1.36, 95% CI 0.56–3.3 for PIRADS). The corresponding Kaplan–Meier curves are provided in the Supplementary Material (Figures S1 A and B).

## DISCUSSION

Focal cryotherapy has demonstrated excellent functional outcomes; however, its oncological efficacy remains under investigation due to limited data on cancer control. In this study, we found that three years following cryoablation, seven out of eight patients remained free of radical treatment, and two out of three were free of any recurrence. Notably, we observed no significant difference in prognosis between patients with GG 1 disease and those with a higher GG at diagnosis.

The impact of FT on urinary and sexual function has been well-documented, with severe complications reported in less than 3% and 6% of patients, respectively. In contrast, RP and RT are associated with urinary incontinence rates of 13% and 4%, and erectile dysfunction rates of 76% and 72%, respectively (2, 17).

All patients with GG1 disease should be counseled to consider active surveillance as the recommended first-line strategy, given its favorable long-term oncological outcomes. However, for selected patients, focal therapy may provide a suitable, minimally invasive alternative.

Given that FT has already demonstrated superior functional outcomes compared to conventional treatments, our study focused on its primary challenge: oncological outcomes.

Follow-up protocols after focal therapy vary widely across studies, impacting the interpretation of oncological outcomes. There is a heterogeneity in biopsy approaches (e.g., number of cores, transrectal vs. transperineal, targeted vs. systematic) and triggers for biopsy (e.g., protocolized vs. based on clinical suspicion such as rising PSA or MRI findings). Recent expert consensus recommends performing an MRI and control biopsy within 6–12 months post-treatment (18, 19). In our protocol, an initial MRI was performed within six weeks to detect complications, followed by a second MRI at 12 months to evaluate potential recurrences before performing a confirmatory biopsy. The median time to biopsy in our study was 18 months, compared to 6, 12, and 24 months reported in other series (20–22).

In our cohort, 24 patients (22%) declined confirmatory biopsy, consistent with refusal rates of 16–23% reported in other studies (20, 23). The primary reason for refusal was low suspicion of recurrence, based on stable PSA levels and negative MRI findings. In the absence of suspicious clinical or imaging features, it is possible that a proportion of these patients would have had negative biopsy results; however, this remains hypothetical due the lack of histological confirmation. Our overall positive biopsy rate of 32% is slightly lower than the rates reported by Baskin, Esaú, and Marra, but significantly higher than the 7% reported by Wysock et al. (20) (21–23). These different cryotherapy cohorts in the literature show that confirmatory biopsy positivity rates in patients with baseline Grade Group 1 (GG 1) prostate cancer vary widely, ranging from 7% to 49%. This variation is influenced by factors such as biopsy technique and follow-up duration, with higher positivity rates observed in studies utilizing more extensive sampling (e.g., 24-core biopsies) and longer surveillance periods. Notably, out-field progression was more frequently observed than in-field recurrence, highlighting the multifocal nature of prostate cancer and the importance of comprehensive biopsy strategies to guide treatment planning.

We performed cryotherapy in 34% of patients with GG1 disease, 65% of whom had MRI-visible lesions. While active surveillance (AS) remains the standard of care for GG1 disease, patients with MRI-visible lesions have a higher risk of AS discontinuation at five years (63% vs. 48% for those with negative MRI) (9). Although intermediate-risk patients are often considered the primary candidates for FT, this recommendation is largely based on expert opinion (8). Our findings suggest that oncological outcomes are comparable between patients with baseline GG1 and GG >1 disease. These results are in line with those of Khan et al. (15), who, in a cohort of 163 patients, also found no significant differences between Gleason 6 and higher-grade disease when using biochemical recurrence-free survival (Phoenix criteria) as the primary endpoint. While our study focused on histological recurrence and the need for additional treatments, the concordance between both studies supports the idea that baseline Gleason score may not substantially influence recurrence outcomes after focal cryotherapy, thereby challenging the notion that GG should limit FT eligibility.

Beyond biopsy findings, biochemical recurrence and the need for secondary treatments have been proposed as early oncological endpoints for FT. A recent systematic review identified Phoenix criteria for BCR, salvage focal re-treatment, and salvage radical treatment as the most commonly used endpoints (24). We did not analyze BCR due to its variable definitions and unproven correlation with more robust endpoints (e.g., biopsy results, clinical recurrence, metastasis) in the context of FT. At three years, 65% of our patients remained recurrence-free. Unlike previous studies that excluded biopsy findings from their recurrence definitions, we propose that any recurrence—including positive biopsies and secondary treatments—provides a more comprehensive measure of treatment failure.

Additionally, 88% of our patients avoided radical treatment at three years. This aligns with findings from Baskin, Shah, and Marra, who reported radical treatment-free survival rates of 96%, 91%, and 88% at two, three, and five years, respectively. Although small sample sizes and varying baseline characteristics (e.g., 76% GG1 in Marra's study vs. 5% in Baskin's) may influence these outcomes, the consistency across studies suggests that FT provides reliable oncological control across diverse patient populations. In our cohort, no significant differences were observed between GG1 and GG >1 groups in recurrence-free survival (HR 1.2, 95% CI 0.6–2.5) or radical treatment-free survival (HR 1.1, 95% CI 0.35–3.2).

In summary, we present short-term oncological outcomes from a cohort of primary PCa patients treated with focal cryotherapy at a single institution. Our findings demonstrate adequate cancer control with this technique at 3 years of follow-up, with no significant differences in outcomes based on baseline Gleason score. However, this study is limited by its retrospective design, which carries risks of selection and information bias, and by its relatively small sample size, which may reduce the statistical power to detect significant differences between Gleason score subgroups. The median time to confirmatory biopsy exceeded the recommended timeframe of 6 to 12 months, according to international consensus, potentially underestimating early recurrences. Additionally, the choice of salvage treatment was not protocolized. Further prospective studies with larger cohorts are warranted to validate these findings and to clarify whether Gleason score should play a role in the indication for focal cryotherapy.

## CONCLUSION

Focal cryotherapy provides effective short-term cancer control for localized prostate cancer, with the majority of patients remaining free from recurrence and radical treatment at three years. Importantly, outcomes were similar regardless of baseline Gleason score, suggesting that cryotherapy is a viable option for a broad range of patients. However, the study's retrospective design and limited sample size highlight the need for larger, prospective studies to confirm these findings and further refine patient selection criteria.

## Data Availability

Data will be available upon request
