# Supplementary material for: Focal Cryotherapy in Prostate Cancer. Does Gleason Impact Results?
Source: Int Braz J Urol. 2025 Dec 20;52(2):e20250289. doi: 10.1590/S1677-5538.IBJU.2025.0289 (PMC13124189; doi:10.1590/S1677-5538.IBJU.2025.0289)
Supplement: Supplementary file 1 [file 1677-6119-ibju-52-02-e20250289-suppl1.pdf]

APPENDIX

Supplementary Figure 1 - Kaplan-Meier curves for recurrence=free survival according to (A) MRI findings and (B) baseline PSA level.

A.

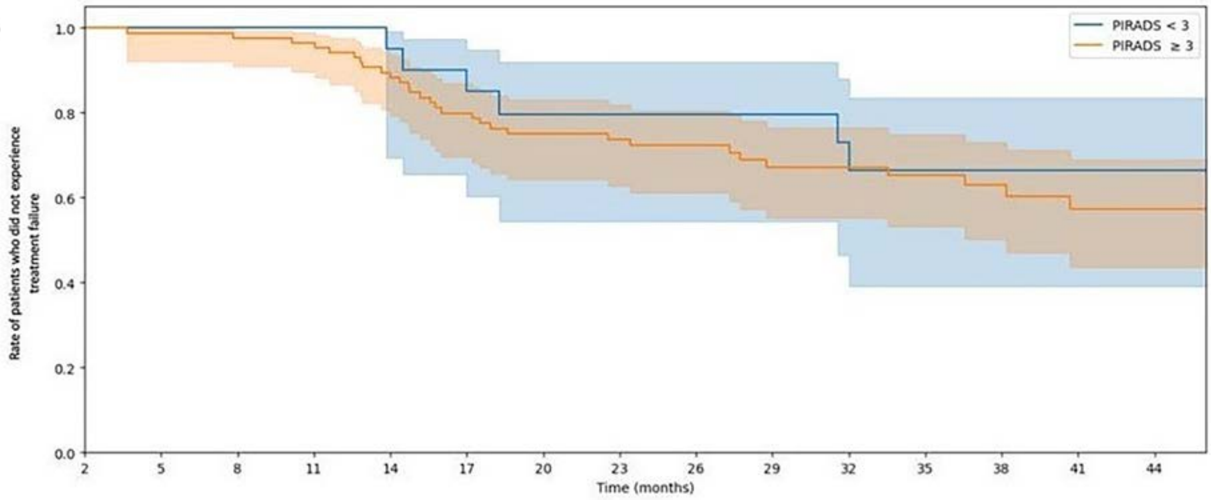

PIRADS < 3															
At risk	22	22	22	22	19	17	15	14	13	12	10	9	9	9	9
Censored	0	0	0	0	2	2	3	4	5	6	6	7	7	7	7
Events	0	0	0	0	1	3	4	4	4	4	6	6	6	6	6
PIRADS ≥ 3															
At risk	88	86	85	83	75	66	58	53	45	39	38	33	24	20	19
Censored	0	1	1	2	3	5	9	13	20	23	24	28	36	38	39
Events	0	1	2	3	10	17	21	22	23	26	26	27	28	30	30

* *PIRADS* - Prostate Imaging – Reporting and Data System

B.

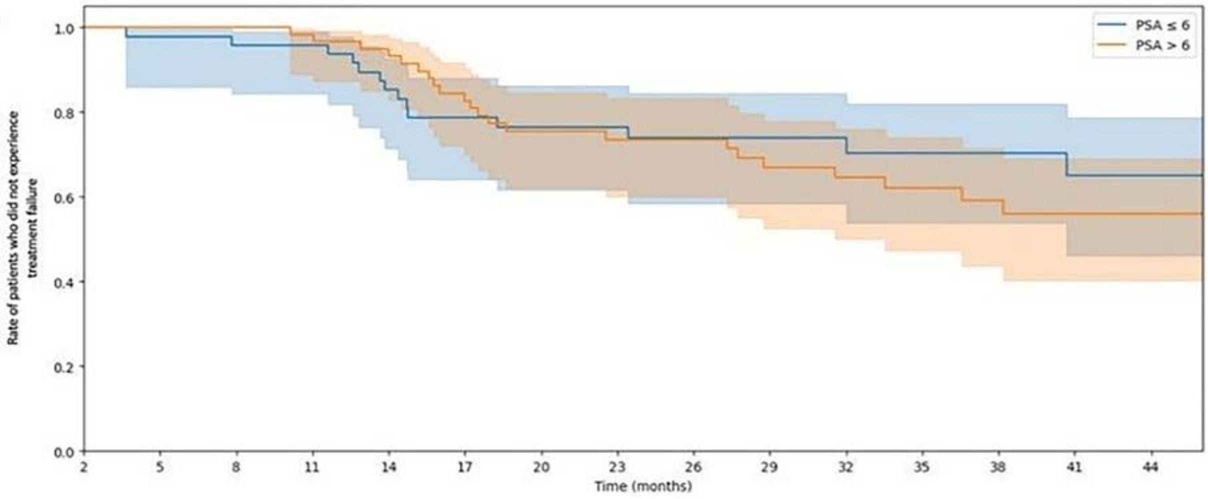

PSA ≤ 6															
At risk	49	47	46	46	40	36	33	30	24	21	20	18	14	12	12
Censored	0	1	1	1	2	3	5	8	13	16	16	18	22	23	23
Events	0	1	2	2	7	10	11	11	12	12	13	13	13	14	14
PSA > 6															
At risk	61	61	61	59	54	47	40	37	34	30	28	24	19	17	16
Censored	0	0	0	1	3	4	7	9	12	13	14	17	21	22	23
Events	0	0	0	1	4	10	14	15	15	18	19	20	21	22	22

* *PSA – Prostate-Specific Antigen*
